# Perspective for genomic-enabled prediction against black sigatoka disease and drought stress in polyploid species

**DOI:** 10.3389/fpls.2022.953133

**Published:** 2022-10-28

**Authors:** Luther Fort Mbo Nkoulou, Hermine Bille Ngalle, David Cros, Charlotte O. A. Adje, Nicodeme V. H. Fassinou, Joseph Bell, Enoch G. Achigan-Dako

**Affiliations:** ^1^ Genetics, Biotechnology, and Seed Science Unit (GBioS), Department of Plant Sciences, Faculty of Agronomic Sciences, University of Abomey Calavi, Cotonou, Benin; ^2^ Unit of Genetics and Plant Breeding (UGAP), Department of Plant Biology, Faculty of Sciences, University of Yaoundé 1, Yaoundé, Cameroon; ^3^ Institute of Agricultural Research for Development, Centre de Recherche Agricole de Mbalmayo (CRAM), Mbalmayo, Cameroon; ^4^ Centre de Coopération Internationale en Recherche Agronomique pour le Développement (CIRAD), Unité Mixte de Recherche (UMR) Amélioration Génétique et Adaptation des Plantes méditerranéennes et tropicales (AGAP) Institut, Montpellier, France; ^5^ Unité Mixte de Recherche (UMR) Amélioration Génétique et Adaptation des Plantes méditerranéennes et tropicales (AGAP) Institut, University of Montpellier, Centre de Coopération Internationale en Recherche Agronomique pour le Développement (CIRAD), Institut National de Recherche pour l’Agriculture, l’Alimentation et l’Environnement (INRAE), Institut Agro, Montpellier, France

**Keywords:** Musa spp., polyploid crops, genomic selection, black sigatoka disease, drought, plant breeding

## Abstract

Genomic selection (GS) in plant breeding is explored as a promising tool to solve the problems related to the biotic and abiotic threats. Polyploid plants like bananas (*Musa* spp.) face the problem of drought and black sigatoka disease (BSD) that restrict their production. The conventional plant breeding is experiencing difficulties, particularly phenotyping costs and long generation interval. To overcome these difficulties, GS in plant breeding is explored as an alternative with a great potential for reducing costs and time in selection process. So far, GS does not have the same success in polyploid plants as with diploid plants because of the complexity of their genome. In this review, we present the main constraints to the application of GS in polyploid plants and the prospects for overcoming these constraints. Particular emphasis is placed on breeding for BSD and drought—two major threats to banana production—used in this review as a model of polyploid plant. It emerges that the difficulty in obtaining markers of good quality in polyploids is the first challenge of GS on polyploid plants, because the main tools used were developed for diploid species. In addition to that, there is a big challenge of mastering genetic interactions such as dominance and epistasis effects as well as the genotype by environment interaction, which are very common in polyploid plants. To get around these challenges, we have presented bioinformatics tools, as well as artificial intelligence approaches, including machine learning. Furthermore, a scheme for applying GS to banana for BSD and drought has been proposed. This review is of paramount impact for breeding programs that seek to reduce the selection cycle of polyploids despite the complexity of their genome.

## Introduction

Several efforts have been undertaken in the improvement of polyploid plants. This improvement involves the creation and the selection of new varieties for the traits of interest and requires crossbreeding in conventional approaches. However, these efforts quickly encountered obstacles because of the complexity of polyploid genomes (Zingaretti et al., 2019). Indeed, the conventional breeding methods appeared to be time consuming and involved enormous phenotyping costs. To select banana varieties for black sigatoka disease (BSD) and drought resistance, the whole selection cycle can take up to 15 years or more. In addition, this traditional breeding is unable to consider all events that occur in polyploid genomes and that significantly influence inheritance traits in the population. Morphological characterization (Jarret and Litz, 1986; Ude et al., 2002; Karamura and Mgenzi, 2004; Nsabimana and Van Staden, 2007) supplemented by molecular studies (Simmonds and Weatherup, 1990; Li et al., 2013) have shown that there is great genetic diversity among *Musa* species, which offers the possibility of implementing genomic selection (GS) on a large population.

GS approach developed by Meuwissen et al. (2001) is capable of selecting a large number of individuals for the traits of interest in a short time, with reduced costs. Because of its potential, GS is considered to be one of the most promising post-1990 approaches in plant breeding, along with genome editing, Quantitative Trait Locus (QTL) mapping, envirotyping, and Genetically Modified Organisms (GMOs) (Haile et al., 2020). The genetic gain of the GS can be two to three times greater than conventional selection. Increasingly, the genotyping-by-sequencing technology allows sequencing the genome of several hundred individuals at lower costs, making it possible to easily apply GS on breeding programs (Poland and Rife, 2012). Today, because of, in particular, advances in high-throughput genotyping, GS is starting to appear in the plant sector where studies have been carried out on plants such as cereals (Crossa et al., 2014), tomato (Picard, 2015), oil palm (Nyouma et al., 2019), rubber tree (Cros et al., 2019; Munyengwa et al., 2021), cassava (de Oliveira et al., 2012), and banana (Nyine et al., 2017; Nyine et al., 2018). As GS is applied to all plants, new approaches are being sought to adapt this tool to the specificity of each genome (Heslot et al., 2015).

Although GS is making progress in diploid plants, it encounters difficulties in polyploid plants due to factors that marker-assisted effect cannot properly control in these species. These factors include allelic dominance, epistatic effects, and the high structuring observed in the germplasm of polyploids. In addition to these factors, the GxE (genotype x environment) interactions have an impact on GS and can be problematic to estimate. Thus, these factors make it difficult to carry out all of the important steps in GS ranging from genotyping to genomic prediction, including the variant calling. Likewise, the initial GS models were developed on biallelic Single Nucleotide Polymorphisms (SNP) approach that considers that the genome of candidates is diploid and the vast majority of variant calling tools are organized in this logic. Applying these approaches to polyploids will *a priori* distort the quality of SNPs and therefore the genetic breeding value of individuals.

GS selection work on polyploids would do better to explore possible solutions to overcome limits encountered so far. Heslot et al. (2015) reported some perspectives for GS in plant breeding including marker-assisted recurrent selection with GS, new phenotyping strategies, efficient selection on multiple traits, and the accommodation of GxE in GS. In this review, we continue in this logical way by taking stock of the status of GS on polyploid plants while exploring new avenues that will allow GS to be efficient in the selection of polyploids. Consequently, we take the banana as a model plant not only because of its diversity at the ploidy level (diploid, triploid, and tetraploid) and its polyploidy nature (allopolyploid and autopolyploid) but also because bananas can reproduce in a clonal and sexual way. First, we present the conventional advances that have allowed the genetic improvement of bananas so far with specific focus on banana breeding for drought- and BSD-related traits. Then, we highlight the limits of GS for polyploid plants, and, finally, we explore how allele dosage, bioinformatics tools, artificial intelligence, consideration of GxE interactions, and genomic composition in bananas can help to implement GS on polyploids with optimal accuracy.

## Advances in banana conventional breeding

### Conventional breeding in the fight against BSD

**Figure 1:**
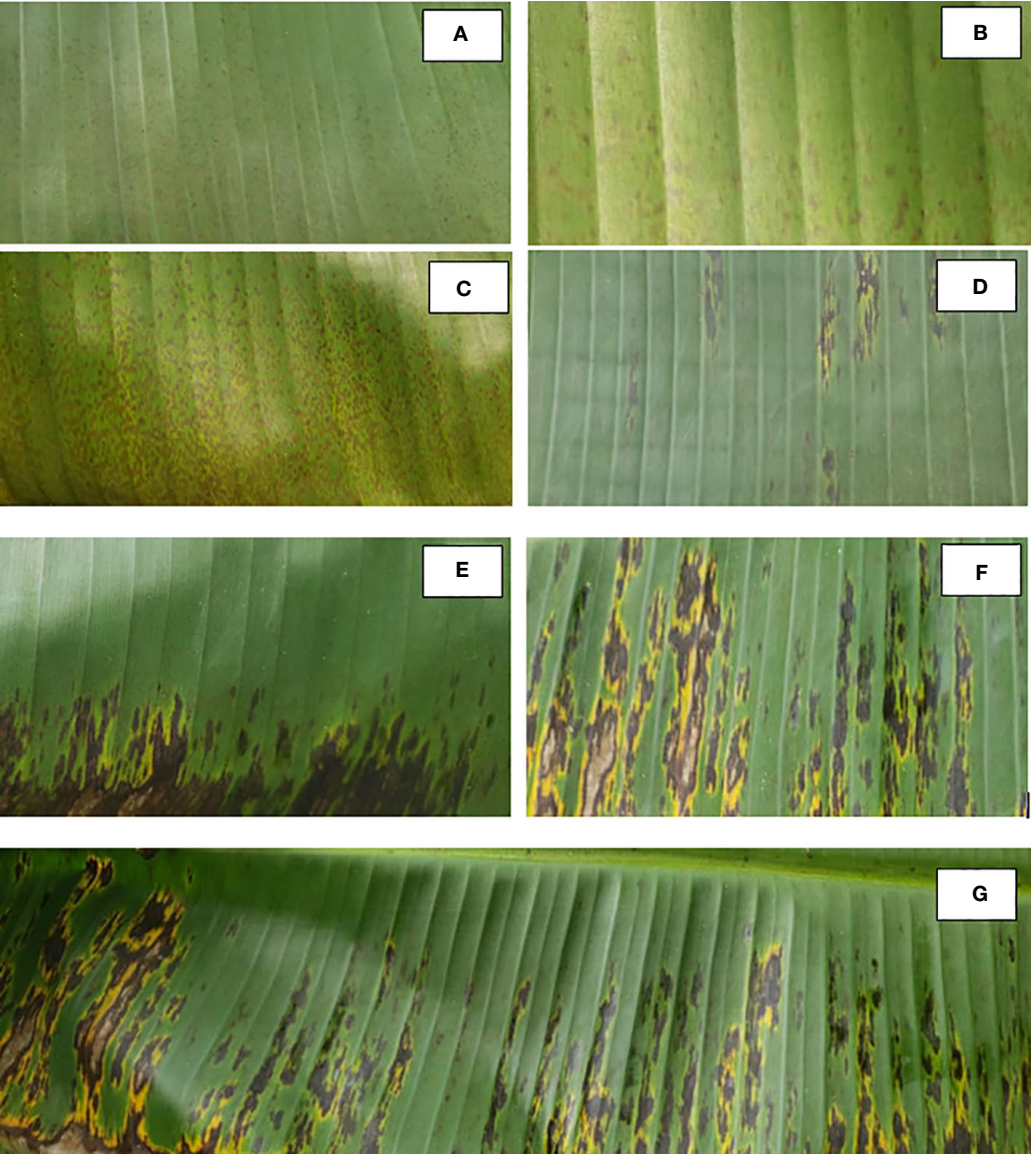
Evolution of black leaf streak disease symptoms observed in the field in the central region of Cameroon (personal observation). **(A)** Stage 1 is the “initial speck stage”. **(B)** Stage 2 is the “first streak stage”. **(C)** Stage 3 is the “second streak stage”. **(D)** Stage 4 is the “first spot stage”. **(E)** Stage 5 is the “second spot stage”. **(F)** Stages 5 and 6 are the “third or mature spot stages”. **(G)** Overview of a sheet where all stages of development of BDS can be observed.

The BSD is considered to be the most destructive among banana leaf spot diseases, and the symptoms of the disease were described in many studies (Fouré et al., 1984; Johanson and Jeger, 1993; Churchill, 2011). According to Fouré et al. (1984), there is a stage before stage 1 reported by classical description, which resembles stage 1 of the yellow sigatoka disease but is distinguished by its punctual and diffuses aspect. Taking this stage into account is essential for disease control, because starting treatments at stage 1 could make eradication of the disease difficult. The first description of BSD was made on Dwarf Cavendish (AAA) and six symptomatic stages reported (**Figure 1**). The dark brown dashes on the underside of the leaves are the first leaf symptoms followed by the appearance of necrosis capable of destroying large leaf areas and causing significant yield losses. In fact, *Mycosphaerella fijiensis* attacks the plant at the leaf level, and young leaves show early symptoms of infection. In principle, BSD does not develop much in dry and high-altitude areas. However, it is increasingly observed that new strains adapt to these hostile locations (Arzanlou et al., 2007).

Numerous intra- and interspecific hybridizations that have taken place within the Eumusa section have contributed to obtaining the species currently cultivated (Li et al., 2013). In the conventional breeding programs, this low capacity of hybridization is used for creating new varieties resistant to the disease. The first attempt to improve bananas date back to 1920, the idea was then to find an alternative to wild cultivars and products of domestication to increase production (Mboula, 2014). These first works were led at the United Fruit Company in Honduras and the Imperial College of Tropical Agriculture in Trinidad; the main improvement objectives were focused on disease resistance, whereas the secondary objectives were concerned with tolerance to abiotic stress (Tomekpé et al., 2000; Mboula, 2014). Today, these objectives remain in breeding programs, and the resistance to BSD is among the most important. The need to extend the parents’ choice to polyploids has faced many difficulties, especially in triploids that are sterile and clonally propagated. Others limitations reported to realize successfully mapping population in banana are cost, space, and disease/biosecurity constraints (Heslop-Harrison and Schwarzacher, 2007). Despite their high sterility, some triploid varieties produce rare unreduced maternal gametes (n = 3x = 33), which, after pollination with a diploid clone, are the source of seeds containing tetraploid embryos capable of germinating (Menendez and Shepherd, 1975; Bakry and Horry, 1992; Hamon and Nicolas, 1997; Ssebuliba et al., 2006).

Through the world, several institutes and research centers are involved in the banana breeding programs for the resistance to BSD. Some working at national level and others at international level. The goal is to offer to farmers a productive plant material and to consumers a quality product. The main breeding programs are conducted in Honduras by the Fundacion Hondurena de Investigation Agricola (FHIA) in Nigeria, by the International Institute of Tropical Agriculture (IITA) in the French West Indies, by the Centre for Cooperation in Agronomic Research for Development (CIRAD) in Cameroon, and by the African Center for Research on Banana and Plantain (CARBAP) and in Brazil L’EMBRAPA-CNPMF (Empresa Brasileira de Pequisa Agropecuaria, Centro Nacional de Pesquisa de Mandioca Fruticultura Tropical) (Hamon and Nicolas, 1997; Mboula, 2014).

FHIA is certainly the oldest of the banana improvement programs, aiming to select improved diploids that can be used to generate edible triploids. It has developed many varieties, the best known of which are FHIA-01, FHIA-02, FHIA-03, and FHIA-21. Varieties FHIA-01, FHIA-02, and FHIA-03 have shown resistance to BSD.

IITA through its centers in Cameroon, Nigeria, and Uganda aims to create valuable quality cultivars. Problems of disease are the main concern with the presence of BSD. In Cameroon, work is particularly concentrated on cultivars of the Plantain group, which is very popular locally (Mboula, 2014).

CARBAP is a research center specializing in sweet bananas and plantains but with a particular focus on the cultivars of the Plantains group. The evaluation of some exotic cultivars in Cameroon showed that the varieties IDN 077, Lagun Vunalir, and Pisang Kelat were particularly resistant to BSD (Noupadja et al., 2001). Dwarf hybrid F568, a second-generation hybrids obtained from the crossing of the tetraploid genotype with an improved secondary diploid developed by CARBAP, distinguished itself from the two other genotypes (C292 another dwarf hybrid and the plantain landrace Red Yad) with a higher number of standing leaves on the plant, suggesting a greater resistance to BSD (Mboula, 2014). Likewise, the hybrid CRBP-39 showed better agro-morphological performances under BSD pressure compared to its parent “French claire” suggesting its resistance to the disease (Cohan et al., 2003). The varieties F568 and C292 were evaluated for BSD in the pedo-climatic conditions of two zones in Cameroon and showed a better production compared to their parent Red Yade (Mboula, 2014).

**Figure 2:**
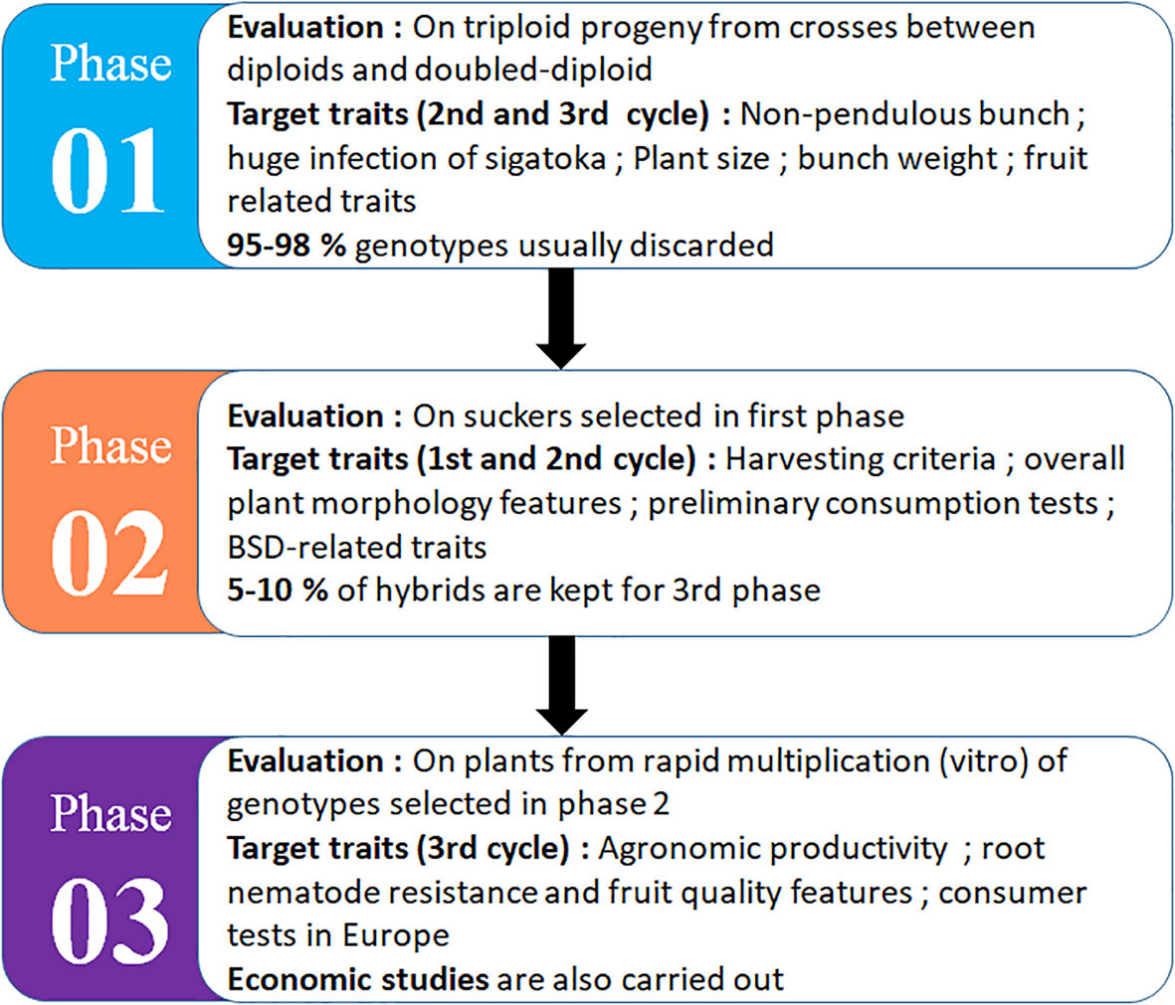
The selection process for triploid dessert export bananas by CIRAD in Guadeloupe [inspired by Bakry et al. (2009)]. The selection cycle can vary from 12 to 15 years depending on the production cycle of the variety: Phase 01 (3–4.5 years), Phase 02 (2–3 years), and Phase 03 (3–4.5 years) for agronomic evaluation, plus consumer test and economic impact assessment.

CIRAD has developed an original improvement strategy based on the creation of triploids from natural or improved diploid plant (**Figure 2**). Located in Guadeloupe, CIRAD’s work aims to produce resistance varieties with quality fruits, because the export market is particularly targeted. Some varieties including FLHORBAN-920, FLHOBAN-918, IRFA-909, IRFA-910, and IRFA-914 were created, but whose diffusion has not always been approved because of morphological and viral constraints.

### Conventional breeding for controlling drought constraint in banana production

**Figure 3:**
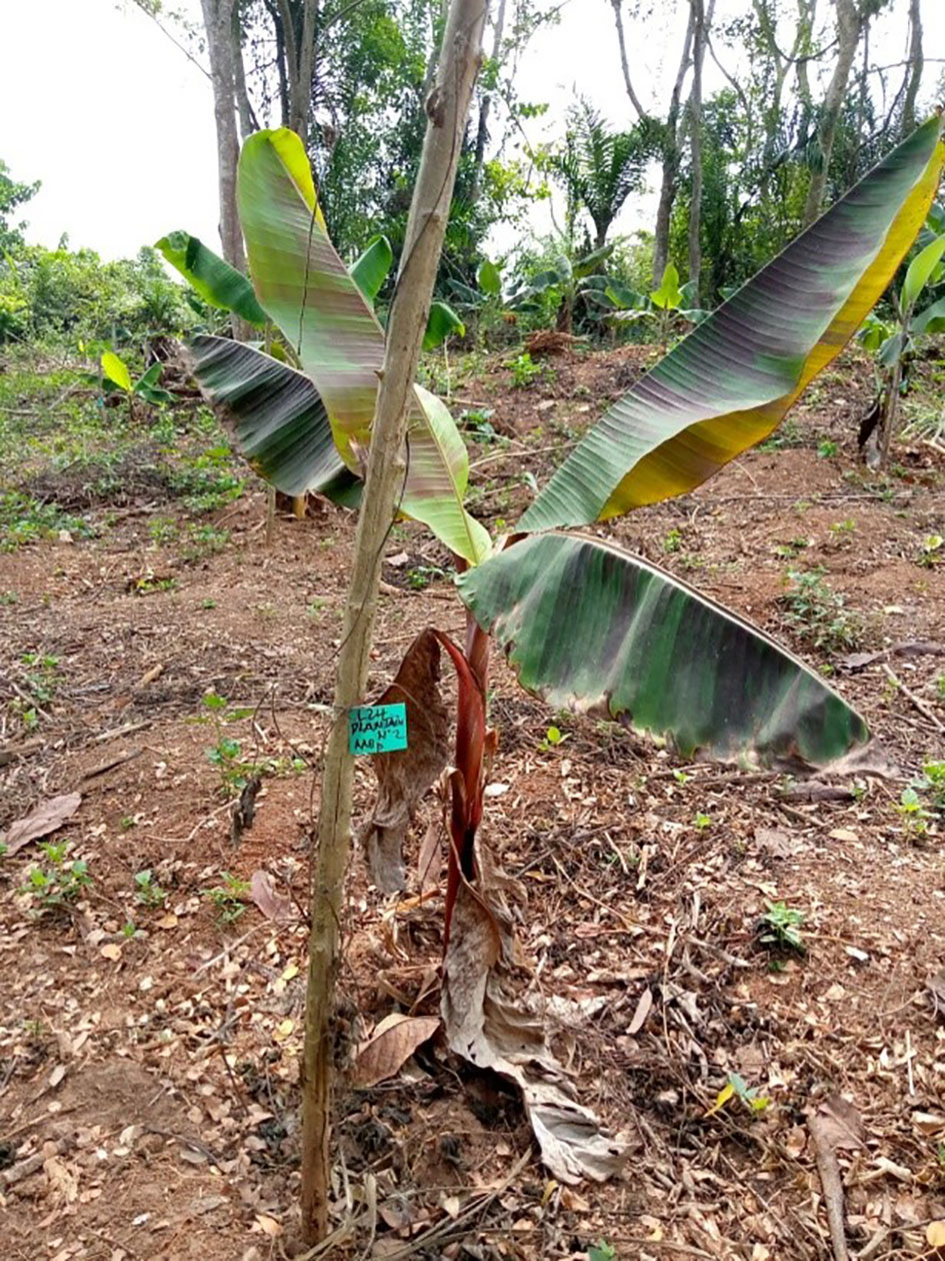
Banana plant (accession plantain N2) affected by water deficit with higher atmospheric temperature in Cameroon. Leaves turn yellow color before wilting if drought persists.

Water is not only an essential nutrient for the plants but also the main means by which plants have access to other nutrients such as nitrogen, phosphorus, and potassium (Brisson et al., 1998; Hu and Schmidhalter, 2005; Surendar et al., 2013). In banana, the water deficit seems to be one of the most important causes of low yield, which results in average weight losses of diet (8%–28%) in dry periods (Ekanayake et al., 1994; van Asten et al., 2011; Kissel et al., 2015). Drought symptoms appear in the form of leaf yellowing and wilting (**Figure 3**). The assessment of banana for drought must cover the morphological, physiological, and biochemical levels as changes occur at all these levels when the plant is stressed (Nansamba et al., 2020; Nansamba et al., 2021; Eyland et al., 2022). Studies have shown that genotypes with low leaf area index, highest root volume, highest water use efficiency, total biomass production, and root dry weight appeared to be drought tolerant (Ekanayake et al., 1995; Megha et al., 2016; Tak et al., 2017b). Panigrahi et al. (2021) pointed out that cord roots are essential in the architecture of the banana plant, implying that cultivars with a high number of cord roots can increase their root size, which leads to the good water and mineral absorption during drought. At the agronomic level, bunch yield is reported to be the trait that better discriminates tolerant genotypes from sensitive genotypes (Ravi et al., 2013). The tolerance of plants to drought depends on genotypes (Ekanayake et al., 1994; Surendar et al., 2013). Genotypes that tend to restrict the opening of stomata in the afternoon have been classified as “water savers”, and this behavior allows them to avoid short periods of water deficit; unlike those whose stomata remain open in the afternoon, this behavior is exploited in conventional breeding. The IITA in Nigeria through the evaluation of triploid and tetraploid cultivars under drought conditions confirmed that the genotypes have different stomatal reaction according to leaf age (Ekanayake et al., 1995). The majority of banana cultivars containing genome B of *M. balbisiana* are more drought tolerant than those solely based on the genome A of *M. acuminata*. This genetic advantage is exploited to incorporate better characteristics into elite varieties in banana. The cultivars Bluggoe (ABB) and Fougamou (ABB) show the greatest differences in leaf conductance between morning and afternoon, and the cultivars Bobby T (ABB) tend to maintain their stomatal conductance throughout the day (Mboula, 2014).

In Egypt, varieties of the AAB and ABB genomic groups showed tolerance to water stress compared to varieties of the Cavendish group (AAA) (Bakry et al., 2009). Similarly, the “Pisang Awak” (ABB) cultivar has shown its ability to adapt to a dry environment in Oman (Bakry et al., 2009). This leads many centers to develop ABB dessert bananas from the AB diploid and *Musa balbisiana* (B) parent. C292 and F568 hybrids (Plantain group) developed by CARBAP were evaluated under drought condition through many regions in Cameroon, and the results allowed to better understand the role of drought in banana plant growth (Mboula, 2014). This study was used as a trigger for the development of varieties resistant to CARBAP. Likewise, in India, recurrent breeding is used by local research centers to develop synthetic diploid strains that are drought tolerant and BSD resistant (Bakry et al., 2009).

Biotechnology tools involving tissue culture, genetic engineering, and, more recently, genome editing technology have, meanwhile, made it possible to overcome the difficulties of crossing in bananas in conventional breeding (Santos et al., 2005b; Pei et al., 2007; Endah et al., 2008; Sels et al., 2008; Mahdavi et al., 2012a; Rukundo et al., 2012; Li et al., 2015; Singh et al., 2015; Zhao et al., 2016; Tak et al., 2017b; Backer et al., 2019a). If the barriers, due to the complexity of the genome, are overcome, then the selection cycle can vary from 12 to 15 years to obtain a variety (Bakry et al., 2009). Nansamba et al. (2020) reported that conventional breeding should combine more molecular and biotechnological tools to facilitate the creation of drought-tolerant banana varieties through the conventional route. The marker-assisted breeding involving GS is explored to reduce challenges in polyploidy crops in general.

### Genomic mechanism of resistance/tolerance against BSD and drought

The mechanism of BSD resistance in banana host is based on the interaction between the major locus (bs) carrying the recessive allele and at least two other genes (bsr) with additive effect (Oiram Filho et al., 2019). These different loci express resistance genes, but their effect is strongly masked by the dominant genes encoding susceptibility in susceptible cultivars. The contribution of ploidy level to BSD resistance has also been reported in the plant–*M. fijiensis* interaction. Indeed, it showed that the major loci controlling parthenocarpy and resistance to BSD and the level of ploidy acted together on growth and yield of banana–plantain. *M. fijiensis* processes tools capable of overcoming resistance in the host when the two interact. Functional orthologs of the Avr4 and Ecp2 genes of *Cladosporium fulvum*, a tomato pathogen, are present in *M. fijiensis* (Stergiopoulos et al., 2014). *M. fjiensis* also produces molecules such as Juglone that can be used by inoculation to assess the resistance of banana cultivars. In addition to the resistance genes, other biological elements may be secreted in abundance by BSD-resistant cultivars. El Hadrami et al. (2005) found high production of active oxygen species including 0_2_^−^ and H_2_0_2_ in the cultivar Fougamou, compared to the susceptible cultivar Grande Naine when subjected to Juglone concentration. To control *M. fijiensis*, the exploitation of transgenic genes is also a pathway that has been shown to be effective in some cultivars. A cultivar “Gros Michel” was transformed by integration of two rice chitinase genes, and results showed a decrease in BSD symptoms by 73%–94% compared to the untransformed cultivar (Kovács et al., 2013). Knowledge of resistance genes in bananas opens a way of selecting cultivars or varieties naturally resistant to drought, according to the quality and quantity of resistance genes expressed. In banana, Nansamba et al. (2020) highlighted the molecular tools that the plant brings into play under water stress. Thus, differentially expressed genes upregulated and/or downregulated are produced depending on the genotype of the cultivar as noticed with drought-tolerant Saba and sensitive Grand Naine. Other elements such as *MusaSNAC1* transcription factors participate in drought tolerance by regulating stomatal closure and hydrogen peroxide production. To identify genes expressed in *M. acuminata* spp. *burmannicoides* var. *Calcutta* 4 (AA) leaves from plants submitted to temperature stress, Santos et al. (2005) found that about 30% of the expressed genes discovered in this specie had been identified in other species. In addition to this, studies aimed at isolating and then inserting the resistance genes in sensitive cultivars are increasingly being carried out (Mahdavi et al., 2012; Backer et al., 2019). The Indian banana cultivar *M. acuminata* cv. *Matti* (AA) transformation by Agrobacterium strain EHA105 has shown that transgenic plants overexpressed salinity-induced pathogenesis-related class 10 proteins from *Arachis hypogaea* AhSIPR10 in comparison to the wild banana, suggesting the role of AsSIPR10 in better tolerance of salt stress and drought conditions (Rustagi et al., 2015). Similar results were obtained by induction of the expression of NAC042 transcription factor in the banana transgenic cultivar Rasthali (Tak et al., 2017).

The knowledge of genomic mechanisms involved in banana plant facing drought and BSD threats is a good perspective for GS, which aims to select varieties based on information carried by the genome. Moreover, the influence of environmental factors in the variations of resistance or susceptibility of banana cultivars underlines the importance of integrating the GxE interaction in GS models.

## Genomic selection for drought and BSD

Projected increases in temperature will result in long periods of drought, which will affect plants, including many polyploids such as roots, tubers, and bananas (RTB) (Friedmann et al., 2018). As a promising breeding tool, GS must pay special attention to the problem of drought to ensure optimal production. Recent studies on GS effectiveness in selecting drought-tolerant varieties have been reported in maize (Beyene et al., 2015; Shikha et al., 2017; Das et al., 2020), chickpea (Li et al., 2018), pea (Annicchiarico et al., 2017), white spruce (Laverdière et al., 2022), etc. Using SNP markers, Shikha et al. (2017) through an evaluation on several environments proved the efficiency of GS for the selection of drought-tolerant maize varieties. Similarly, the effectiveness of GS compared to conventional breeding methods has been proven in maize (Beyene et al., 2015). Laverdière et al. (2022) proposed that drought response in white spruce could be taken into account in multi-trait improvement and then apply GS to candidates at the juvenile stage. GS can also be applied on higher cycles after selection on lower cycles for drought as proven in tropical maize (Das et al., 2020). Apart from the work of Nyine et al. (2018), so far, very little work on GS has considered BSD in banana breeding. Nevertheless, work has revealed the genomic potential of certain cultivars to resist the disease through resistance genes that the plant activates in the *Musa*–*M. fjiensis* pathosystem (Ebimieowei and Wabiye, 2011). RE et al. (2016) reported that the cultivar Calcutta 4 may have resistance genes that recognize PfAvr4 proteins identified in the BSD causal agent.

## Genomic selection in polyploid crops

The advances in next-generation sequencing (NGS) have allowed sequencing many genomes and the first sequencing performed on *Arabidopsis thaliana* (Pelletier et al., 2007). In plant breeding, marker-assisted breeding including GS and QTL-based marker-assisted selection use these technological advances to reduce the selection cycle of varieties, which also leads to the reduction of costs associated with phenotyping (Umber et al., 2016).

GS is based on a dense marking of the genome by molecular markers and the use of specific statistical methods to select individuals. In general, the mixed model or Bayesian methods are capable of simultaneously valuing the information of all markers to estimate the genomic estimated breeding value (GEBV) of the candidates for selection (Xu et al., 2020). The effectiveness of the prediction can be measured by the accuracy of selection, which corresponds to the Pearson correlation coefficient between the GEBV and the true breeding value ( *r*_*TBV*,*GEBV*_) . This accuracy comes into play in the breeder’s equation:


$$R=ir_{TBV,GEBV}\;\sigma_{a}$$

where *R* is the genetic gain over a generation, *i* is the selection intensity, *r* is the selection accuracy, and σ_a_ is the additive genetic standard deviation.

In practice, the GS accuracy is strongly influenced by several factors that deserve to be well managed. These factors include particularly the training population (TP) size and structure, the linkage disequilibrium (LD) in the population, the heritability of traits, the type of marker and marker density use for genotyping, and GxE. Although, today, it is possible to successfully manipulate these factors for the GS in diploid plants, their implementation in polyploid plants remains a challenge due to the complexity of their genome.

## Constraints of genomic selection in polyploid crops

### The difficulty of obtaining a good training population

In banana, the sterility of triploid cultivars makes it difficult to obtain a large related population from hybridization. The study of crossbreeding in East African highland bananas (*Musa* spp. AAA) showed that 72% of the seeds appeared normal and only 59% contained embryos, of which 9% (range, 0%–22%) germinated (Ssebuliba et al., 2006). Under these conditions, obtaining a large TP in banana is not easy. In particular, it will be difficult to obtain the type of TP that is generally used for GS, such as a breeding population or a set of biparental families. As a consequence, the only possible option is to use a germplasm collection or a mixture of germplasm collection with unbalance between different populations. The risk that a germplasm collection is structured is high; it is then mandatory to study the structure of such population to eliminate certain individuals and obtain a less structured and/or more balanced population. However, Nyine et al. (2017) conducted a study of the variation of traits on a population of more than 300 individuals and were able to obtain a TP among related individuals in the population. This study, therefore, opened up a hope of obtaining a good TP in bananas and implementing GS.

### Polyploids have a complex genome difficult to handle

The complexity of polyploid genomes hides much genetic information that could certainly influence the accuracy of selection in GS. This complexity is explained particularly by the poor knowledge of the behavior of chromosomes during meiosis, by a high level of allele dosage and genotypic class compared to diploids, and by the possibility of multivalent pairing (de Bem Oliveira et al., 2019). The complexity of polyploidy genome also explained that many plants including bananas, sugarcane, potato, blueberry, and strawberry are clonally propagated. The majority of cultivated bananas reproduce vegetatively, and they have very low reproduction. These qualities make any genetic improvement on banana constraining and require a long process (Sardos et al., 2016). Vegetative propagation in banana as in many polyploids involves prolonged mitotic propagation. However, prolonged mitotic propagation can result in the accumulation of mutations that cannot be expelled and identified in the population, thus altering the gene pool (Jankowicz-Cieslak et al., 2012).

Likewise, clonally propagated plants usually that have a limited population structure, various origins, extensive outcrossing, a long cycle in the juvenile phase that lengthens the time of phenotyping, and widespread hybridization have also suffered from mild domestication bottlenecks due to clonal spread (Miller and Gross, 2011). The main challenges for the implementation of GS on clonally propagated crops include the modeling of genetic effects and non-additive effects, LD between markers and quantitative trait loci, size of TP, and the number of generation following the training model (Varshney et al., 2017). In addition, the genetic architecture of the traits influences the accuracy of GS.

### Non-additive effects are important in polyploidy crops

An important aspect in genomic predictions is the consideration of non-additive effects such as dominance and epistasis. These non-additive effects are less frequent in sexually propagated plants and diploids, and the majority of GS models consider them to be residual effects. In banana, the influence of epistasis effects on fruit size and weight in some progenies was identified as significant in plantain (Ortiz et al., 1997). The same study reported significant epistasis interactions between the additive and non-additive effects of genes involved in BSD resistance and the level of ploidy for plant height, girth, and fruit size. This explains the need to take into account these non-additive effects in genomic predictions in banana.

However, in clonally propagated polyploids, the dominance and epistatic effects are important and their impact in GS is proven (Gouy et al., 2013). Likewise, heritability in the broad sense is a key factor affecting the accuracy of GS; its estimation is made on the basis of additive genetic variation, which works for sexual plants and not for clonally propagated crops. For asexual plants, it amounts to using heritability in the broad sense, which then implies taking into account the dominance, additive, and epistasis effects (Varshney et al., 2017). Dominance in polyploids can be heritable and thus has an impact in the prediction, making that its estimate should not be neglected (Amadeu et al., 2020). Thus, Amadeu et al. (2020) showed in the tetraploid potato that even if the accuracy of GS was similar depending on whether they considered the dominance effects in the model or only the additive effects, the dominance effects partly explained the variation of GEBVs.

### The variant calling is restricted by non-adapted platforms

GS is a tool that requires both molecular and phenotypic data. Genotyping for polyploidy species has always encountered enormous difficulty because the first genotyping platforms were developed for diploid species, which are not suitable to polyploid plants. If the discovery of SNPs is relatively accessible in single genomes, then it is restricted for polyploid genomes (Mammadov et al., 2012). The important problem in polyploids is that they do not have a reference genome for sequencing, because, generally, their parents are diploids (Matias et al., 2019). In the latter, the allele markers may be of different dosage, which must then be clearly followed when calling SNPs; otherwise, the effects of certain markers will be ignored, and this may impact genomic predictions. An important amount of cultivated bananas harbors the two wild genomes, A and B, of *M. acuminata* and *M. balbisiana*. The call for variants must take into account this aspect and highlight the SNPs common to these two species.

Although NGS today makes it possible to sequence complex genomes, the detection of SNPs in polyploids is also constraining to differentiate true SNPs from paralogous variants, which is explained by the high similarity between the homologous and homeologous sequences (Zingaretti et al., 2019). Under these conditions, it is important either to better adapt the tools used in diploids to polyploids or to develop specific platforms for variants calling.

### The strong GxE interaction influences the accuracies in GS

The occurrence of GxE interactions in plant breeding has always been a challenge to measure the actual impact of genes on quantitative trait variation. In GS, this interaction is essential because candidates are selected on the basis of information contained in the genome, whereas traits like drought- and BSD-related traits are strongly influenced by the environment. BSD and drought constraints are directly accelerated by the environmental factors such as temperature, humidity, and soil components. Polyploids have a high adaptability that gives them potential stability in different environments. However, in the face of constraints such as drought and BSD, this adaptability can vary, and it would then be important to incorporate the environmental effects associated with these constraints in prediction models. It is at this level that genomics could play an important role in making it possible to detect variations at the genome scale due to environmental effects (Prohens et al., 2011). For polygenic traits in complex genomes such as found in polyploids, the occurrence of GxE interactions is also expected to be very difficult to manipulate compare to diploid. It will then be necessary to evaluate the TP over several environments and over several seasons to hope capturing these interactions (Varshney et al., 2017).

## Perspectives to succeed GS on polyploid crops

**Figure 4:**
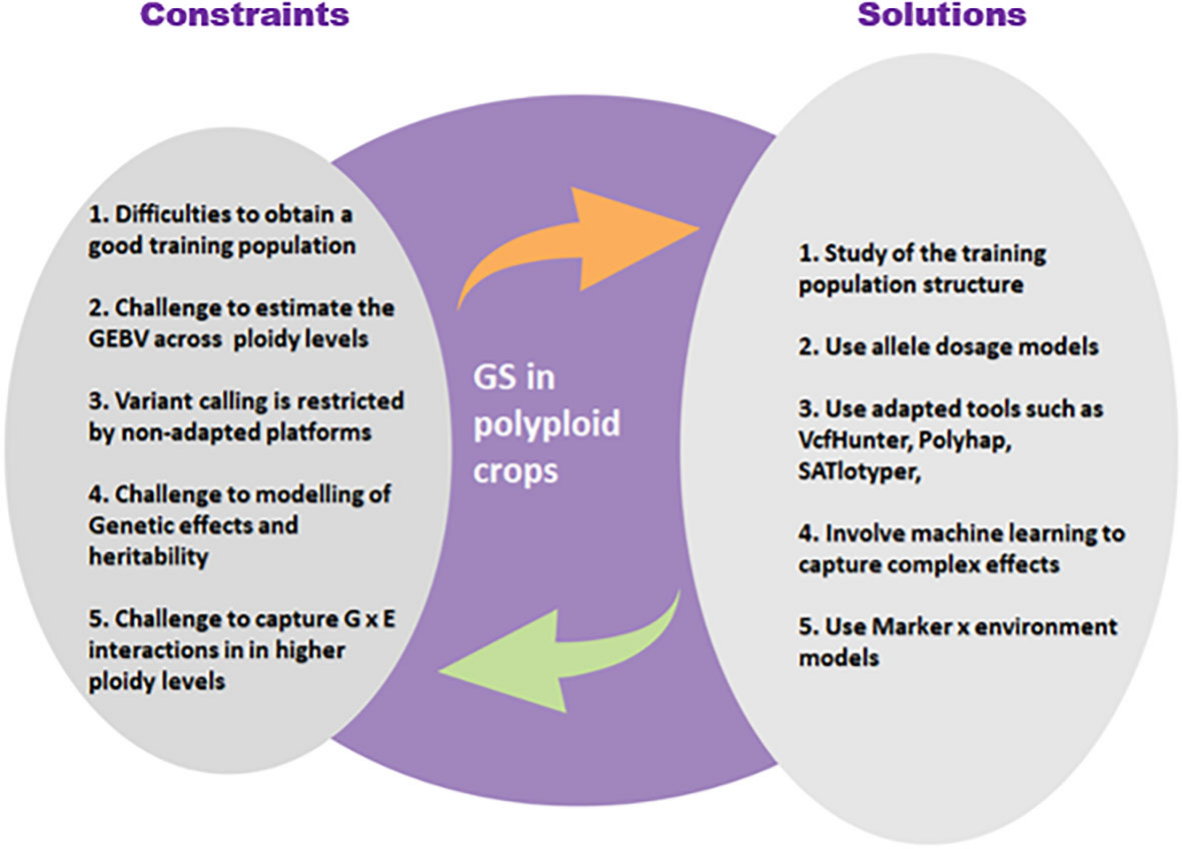
Main constraints of the application of GS in polyploid plants and possible solutions to these constraints.

Several alternatives and solutions are available to the breeder today for working effectively on polyploid crops despite the complexity of their genome (**Figure 4**).

### Genotyping using appropriate tools

During the genotyping process involving NGS, the raw sequence data obtained must be processed not by platforms that call SNPs according to the diploid models but rather by tools suitable for polyploid genomes. In the specific case of banana, it will be necessary to see to what extent the partial or total inclusion of genomic information from one or the other of the parents influences the quantity and quality of the variants obtained and then the prediction accuracy. To better adapt the variant calling and the manipulation of the polyploid genome, several platforms have been specifically designed (**Table 1**). VcfHunter specifically performs variant calls, taking ploidy level into account. This tool has contributed to several genomic studies in bananas (Baurens et al., 2019; Martin et al., 2020; Biabiany et al., 2022) and sugarcane (Garsmeur et al., 2018). VcfHunter in two stages of prefiltering and filtering is able to produce a good quality molecular dataset based on the fastq data obtained from genotyping. In addition, Voorrips et al. (2011) reported fitTetra package of R language as an algorithm capable of performing SNP calling in tetraploids using the tetraploid potato as model plant. Studies performed on plants with high ploidy level proved that one better way to incorporate the polyploid component into the genomic-enabled prediction was to implement the allele dosage (Nyine et al., 2018; Lara et al., 2019; de Bem Oliveira et al., 2019). The allele dosage is an integrated concept in GS especially for polyploid crops that allows to see what level of ploidy and for which trait or group of traits the selection accuracy is better. In bananas, Nyine et al. (2018) noted that taking into account the allele dosage in the model reduced the predictive ability to 15% compared to the traditional biallelic SNP marker model in all statistical models used, with the decrease depending on traits. However, when genotypes of two ploidy levels were used to form the model and only genotypes of the same ploidy level included in the test set during cross-validation, the allele dosage positively increased the accuracy of all fruit filling traits in tetraploids. The results obtained in *Panicum maximum*, an autotetraploid herbaceae, seem to show that the application of the allele dosage gives results that depend on the crop or the ploidy level (Lara et al., 2019). Thus, the allele dosage comparing the GS-TD (triploid dosage) and GS-DD (traditional diploid dosage) models has shown that the GS-TD yielded higher predictive abilities than GS-DD data, suggesting that the traditional assay with diploid dosage will bias the prediction of the six traits evaluated in the species. Using genetic and genetic + dominance effect models, Batista et al. (2021) applied allele dosage in GS on sugarcane and sweet potato. They expanded the method used for GS in autotetraploids and showed that allele dosage can help improving GS in polyploids crops when the frequency of heterozygous genotypes in the population was low.

**Table 1:** Overview of different bioinformatics programs and packages used for genomic in polyploids.

Tools	Case of study	Ploidy level	Application	Reference
**VcfHunter**	Banana	Any	Variant calling and population mapping	(Martin et al., 2020)
**polyHap**	–	Any	Haplotype phasing	(Su et al., 2010)
**SATlotyper**	Potato	Tetraploid	Handle polyallelic SNP data	(Neigenfind et al., 2008)
**randomForestin R language**	*Urochloa* spp.	Any	Missing data imputation	(Matias et al., 2019)
**PolyRAD**	Rdata	Any	Imputation and genotyping	(Clark et al., 2019)
**fitTetra in R language**	Potato	Diploid and tetraploid	Genotype calling	(Voorrips et al., 2011)
**BeadarrayMSV**	–	Diploid and tetraploid	Genotype calling	(Grandke et al., 2014)
**Supermassa**	Potato and sugarcane	Any	Genotype calling	(Serang et al., 2012)
**Outcrosseq**	Sweet potato	Any	Genotyping, haplotyping, and imputation	(Jubin et al., 2021)
**PopPoly**	Tetraploid potato	Any polyploid	Imputation, haplotyping, and genotype dosage estimation	(Motazedi et al., 2019)
**PedigreeSim**	Simulated population	Diploid and tetraploid	Simulation of meiosis	(Voorrips and Maliepaard, 2012)
**pSBVB**	Simulated population	–	Predictions on polyploids	(Zingaretti et al., 2019)
**Polylink**	–	Polyploids with 2, 4, 6, 8, and 10 ploidy levels	Linkage mapping	(He et al., 2001)
**TetraploidMap**	–	Tetraploid	Linkage mapping	(Hackett et al., 2007)
**PolySegratio**	Simulated population	Any	Segregation ratio analysis	(Baker, 2008)
**Polycat**	Cotton	Any	Subgenome categorization	(Page et al., 2013)

### Use adapted tools for impute missing data in polyploidy

The dataset obtained after variant calling usually contains missing data that must be imputed. In polyploids, imputation of missing data encounters the same difficulties as variant calling and requires the use of specific tools. In banana, libraries implemented in the R software were used by Nyine et al. (2018) to impute missing data with acceptable results. Polyploidy explains complex multi-allelic copy number variations (CNVs). polyHap is a software that has been found suitable for imputing missing SNPs in polyploids (Su et al., 2010). polyHap was initially found to be effective in detecting duplicated regions of the genome and thereby seeing CNVs associated with disease in humans. According to Su et al. (2008), polyHap has shown superior accuracy compared to SATlotyper, another program that infers missing data in polyploid genomes, which demonstrated its ability to achieve a good imputation in the tetraploid potato (Neigenfind et al., 2008). Likewise, PopPoly is a method for obtaining haplotypes in polyploid organisms and used in missing genotype calling. Compared to conventional methods, it showed better performance on the tetraploid potato (Motazedi et al., 2019). Another tool called PolyRAD, an R software package, considers the ploidy level of genotypes during variant calling. Its effectiveness in terms of imputation of polyploid genomes was reported when the structure of the population is taken into account (Clark et al., 2019). Outcrosseq software was developed to contribute in mapping on heterozygotes. One of its algorithms called “autopolyploid-plant” is specialized in the imputation of SNP data. However, its imputation accuracy proved to be superior to that of PolyRAD and PopPoly on a simulated dataset and on sweet potato data (Jubin et al., 2021). To get the best out of polyploids, it would therefore be wise to conduct a comparative study of these programs and select the one that gives the best results.

### Artificial intelligence approaches for overcoming the complexity of polyploid genome

**Figure 5:**
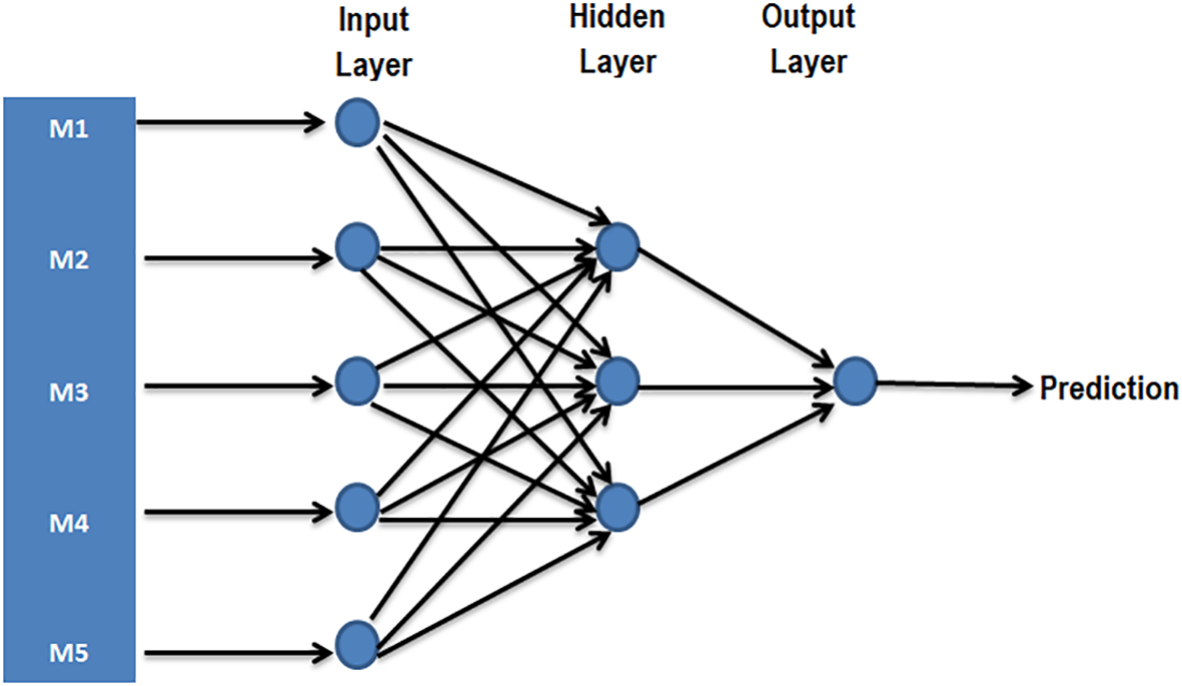
A simple case possibility of prediction with neural network with a single hidden layer [inspired by McDowell (2016)]. As part of GS, the input layer represents one neuron for each molecular marker (SNP markers, for example, M1, M2, M3, M4, and M5), whereas the output represents single neuron combining information necessary to predict the phenotype or BLUP.

The challenge linked to large-scale phenotyping of the population as well as the analysis and interpretation of large datasets are now facilitated by new tools of artificial intelligence. Among the artificial intelligence tools that can be used in GS, machine learning (ML) has already been reported to have enormous potential especially for complex organisms. ML is an artificial intelligence program designed to simultaneously process data for several parameters using associated algorithms. The implication of computer science that uses algorithms and existing samples to capture the characteristics of well-defined patterns in ML is thus a great advantage in the context of GS in polyploids (González-Camacho et al., 2018). In general, the random forest and the support vector machine are the widely used methods in ML (Montesinos-López et al., 2021). The classic GS approaches use the parametric methods that assume that the inputs are normally distributed, which is not always the case, and the input data must then be arranged, whereas the ML takes into account each type of input and includes the epistatic effects. Artificial neutral networks used as ML tool in GS consists of a series of interconnected neurons that map inputs to outputs (**Figure 5**). By comparing the classical approaches Bayesian lasso (BL) and Bayesian ridge regression (BRR) with the neural network, McDowell (2016) showed that the use of neural network method significantly improves the accuracy of prediction compared to the classical BL and BRR methods.

In addition, deep learning (DL) is a set of non-parametric ML methods known to be adapted on situations with very large complex inputs and with complex patterns as expected in polyploids. Zingaretti et al. (2020) obtained on strawberry that DL prediction was not better than linear models except when the epistasis is important. In addition, Ma et al. (2018) compared DeepGS, a DL method that uses the convolutional neural network, with the conventional ridge-regression best linear unbiased prediction (RR-BLUP) method to predict phenotypes from genotypes and found that DeepGS could be complementary to RR-BLUP. Montesinos-López et al. (2021) mentioned the main reasons why DL is good for GS, namely, the capturing of complex pattern in the data, DL supports varied input (raw input, genomic data, environmental data, and omics data), DL can easily handle large datasets compared to classical statistical methods, and its network permits a lego-like construction of new models. Another possibility for using DL in polyploid prediction is the use of hidden Markov models (HMMs) whose potential has already been proven in temperature and energy predictions (Joshi et al., 2017; Ghasvarian Jahromi et al., 2020). HMMs in DL are extensions of Markov chains used to manage missing or hidden data. Joshi et al. (2017) were able to predict with accuracies of up to 19% and 12% the maximum and minimum temperatures, respectively, to mitigate the risk of snow avalanches in the Himalayas. Thus, the meteorological data used could be replaced by phenotype data on BSD- and drought-related traits. Similarly, Khadr (2016) showed that HMMs gave a fairly good result between observed and predicted precipitation, concluding to the effectiveness of these models in managing drought in the Blue Nile river basin, Ethiopia. HMMs have also proven to be effective in predicting complex plant phenomena. Tseng et al. (2020) were able to predict with an accuracy of 83.3% the yearly formation of birch pollen that is a difficult phenomenon to manage in the plant kingdom. Similarly, N-terminal myristoylation, which plays a crucial role in the response of plants to environmental stresses such as drought, could be predicted using an approach integrating HMMs (Podell and Gribskov, 2004). However, DL is a very sophisticated technology whose implementation requires adaptation in the context of GS.

### MxE models to handle the GxE interaction

Although polyploids have the potential to adapt to multiple environments, GS models must integrate the MxE (marker x environment) interaction so as not to bias the predictions. Lopez-Cruz et al. (2015) underlined the importance of MxE models in genomic prediction: They are models that can easily be implemented; more importantly, they decompose the effects into two components, one of which is common to all environments and the other specific to each environment. This enables to trace the regions of the genomes that are responsible for the variations observed on different environments and those that are stable; this information is ignored by standard multi-environment models. Jarquín et al. (2014) used covariate function in the model to overcome the issue related to GxE interaction in GS on wheat and obtained a higher prediction for the models including interaction factors compared to the models based on marker effects only. The GxE models take account of environmental conditions such as temperature, soil type, humidity, radiation, and precipitation. Likewise, BGGE (Bayesian genomic genotype x environment interaction) is a package used in the R software developed to incorporate MxE effects into the calculation of genetic values (Granato et al., 2018). In genomic-enabled prediction, BGGE can be used in single or multiple environments. Another approach is to integrate environmental covariates into GS to predict GxE deviations in unobserved environments (Grattapaglia, 2017). However, it is important to handle these MxE models well because they have the disadvantage to impose a scheme for estimating GxE effects but are suitable for analyzes where environments are positively correlated.

### Effect of banana genome on TP size and structure

The diversity at the ploidy and genomic level of the banana is an aspect that deserves to be explored in depth because it could have a great impact on the efficiency of GS. If it is obvious that the allele dosage approach in banana depends on the ploidy level as reported by Nyine et al. (2018), then it is also noted that the genomic composition of heterozygous individuals can provide information on the influence of different banana genomes (A and B) in the predictive ability obtained by allele dosage. The predictive ability obtained by allele dosage on homozygous individuals with the single genome A or B is probably different from those obtained in heterozygous with both genomes A and B. Taking into account data including all genomes will certainly offer several possibilities for cross-validations (**Table 2**). It will then be a question of seeing how heterozygotes can predict the performance of homozygotes over time (intercycle validation) and in space (intersite validation) and *vice versa*. However, most GS studies have focused on species with the same level of ploidy and on a population from the same species. In banana, cultivars are derived from two parental species, *M. acuminata* and *M. balbisiana*, which make the population structure complex and require understanding. Thus, the high-throughput genotyping required in GS to generate the SNPs necessary for a study of population structure must always involve the reference genomes of both *M. acuminata* and *M. balbisiana*. According to Isidro et al. (2015), the best way to optimize TP seemed to depend on the interaction between population structure and trait architecture. In banana, Nyine et al. (2018) used 77 different families including both species. The results obtained were promising but did not clearly show the role of each species in the predictions. Furthermore, they attributed the inconsistency of the results obtained with the Reproducing Kernel Hilbert Space (RKHS) method on different traits to the complex structure of the TP, which is probably influenced by the presence of two parental genomes.

**Table 2:** Scenarios to study the effect of genomic group composition on GS accuracy of banana triploid cultivars phenotyped for drought- and BSD-related traits.

Scenarios	Cross-validation	Training population	Validation population
AAB→AAB	Intragenomic group	AAB	AAB
AAB→ABB	Intergenomic group	AAB	ABB
ABB→AAB	Intergenomic group	ABB	AAB
AAB→AAA	Intergenomic group	AAB	AAA
AAA→AAB	Intergenomic group	AAA	AAB
AAA→ AAA	Intragenomic group	AAA	AAA
ABB→AAA	Intergenomic group	ABB	AAA
AAA→ABB	Intergenomic group	AAA	ABB
ABB→ABB	Intragenomic group	ABB	ABB

Furthermore, if several studies have shown that selection accuracy increases with TP size before reaching a constant value (Cros et al., 2019), then there is no ideal TP size for a species or for a trait as reported by Bassi et al. (2016). Thus, the ideal remains to use the maximum TP size, vary this size in the model, and determine the one that gives the best accuracy. However, the fact that the banana populations are from two species may increase the genetic structure of the TP and the interaction between TP size and structure could play an important role in prediction accuracy. In comparing TP optimization strategies, Berro et al. (2019) pointed out that there was a trade-off between population size and other factors such as TP structure. Therefore, it is important to combine these two factors in models for a population with a complex pedigree such as the banana population from both species.

### Design of the training population from germplasm and clonally propagated crops

**Figure 6:**
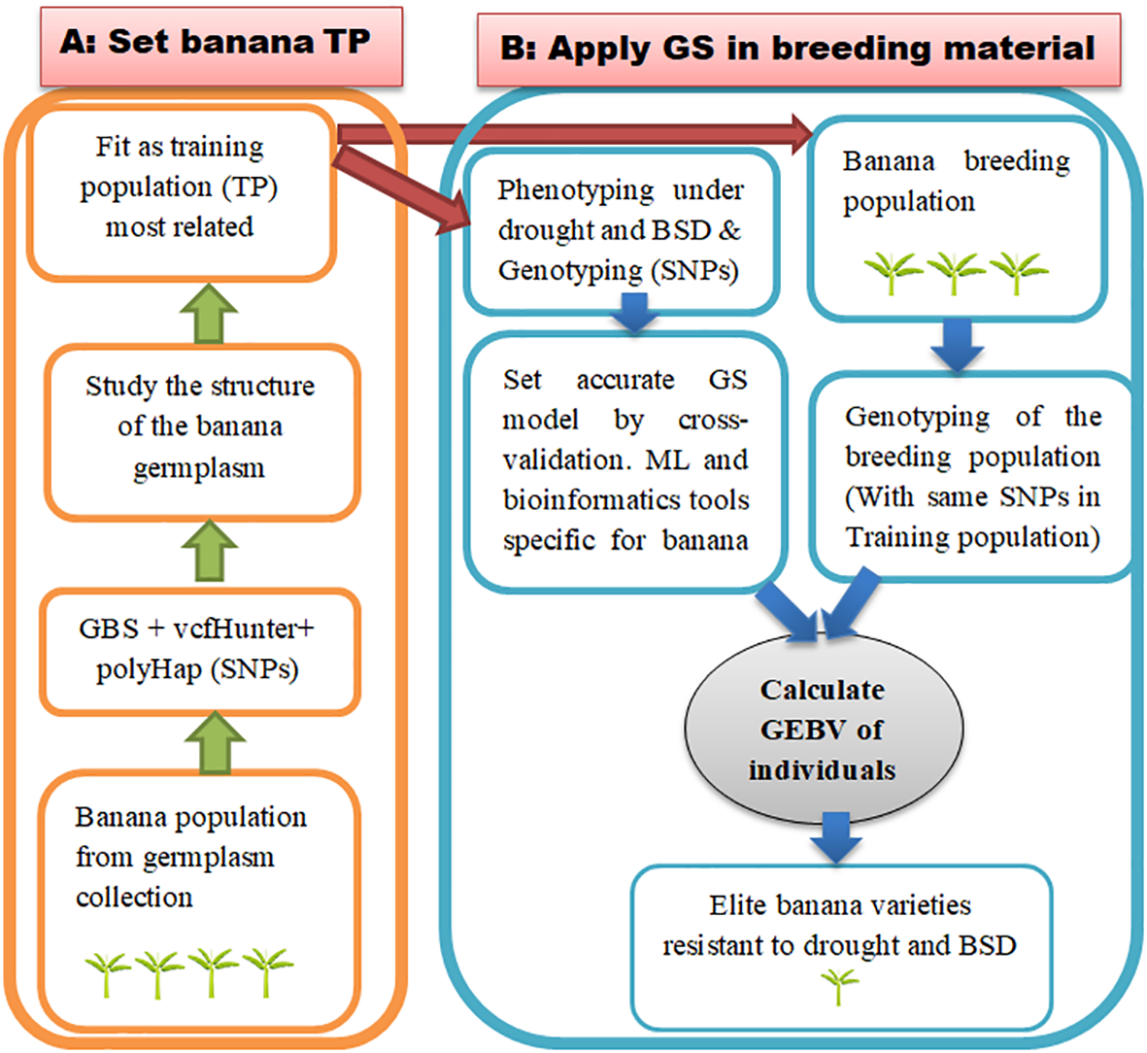
Application diagram of GS on banana population from germplasm collection for drought and for BSD. **(A)** Set a training population from germplasm collection. This involves selecting the most related individuals in the population and using them as training population **(B)** Design an accurate GS model by cross-validation using data from genotyping and from phenotyping for drought and BSD and then implement GS on banana breeding material. This involves calculating the genetic values of individual candidate to selection (breeding material) using the most accurate model. TP, training population.

The recommended TP to optimize the selection accuracy is that with a related population and large size. Varshney et al. (2017) compared the accuracy of selection according to the kinship link between the individuals contained in the TP and proved that 375 half-sib individuals were required to achieve the same accuracy obtained with 50 full-sib individuals. In GS studies, the evaluation of the effect of TP aims to combine these two aspects. Several species are difficult to develop related populations by crossing but present a large number of individuals in the germplasm. For polyploid plants such as banana, another GS approach consists of working on germplasm by studying the structure of the population to group most related individuals together in TP (**Figure 6**). The great genetic diversity in the genus *Musa* is found mostly in natural population found in the banana germplams. This important diversity assumes that obtaining an ideal population for GS is possible. The ideal in GS is to have a wide diversity within the population for the traits of interest because precision is obtained when the phenotypes and genotypes to be tested are within the same range of variation (He and Li, 2020). Nyine et al. (2017) concluded in a diversity study of East African Highland banana and its progeny that the population was genetically diverse. This population was the subject of a GS study with encouraging results (Nyine et al., 2018). In wheat, Haile et al. (2020) reported that it was possible to extract the useful alleles in the germplasm population. For this purpose, prediction error variance (PEV) and generalized coefficient of determination (CD) methods have been reported to optimize the TP from germplasm collection. Hence, PEV is a function that quantifies the prediction errors in the model. PEV therefore evaluates the ratio of these errors to the number of times an individual is measured, the number of relatives of individual, the strength of relationship, and the genetic variance. CD is considered to be the amount of variation in true contrast of the genetic values by contrasts of the predicted genetic values. Another optimization approach is the main component-based that consists in selecting individuals from the population of germplasm collected on the basis of another well-targeted population and not on the basis of kinship within the population.

### Applying GS for drought- and BSD-related traits on bananas

An implementation of GS for drought and BSD in banana is not yet clear to our knowledge. The study carried out to date is that of Nyine et al. (2018), which only integrated BSD-related trait under natural conditions. Drought and BSD, however, are the major threats that drastically reduce yield in banana production. GS should be able to benefit breeders of banana varieties resistant to BSD and drought. As the implementation of GS requires efficient phenotyping and dense genotyping, it will therefore be necessary as a priority to apply this to a banana population subjected to water stress and to BSD. The GS for drought-related trait has already been performed on many species as reported previously with promising results. Although this still seems unrealized in banana, it is evident that banana phenotyping for drought is known and the target traits are generally leaf emergence rate, cell membrane stability, stomatal conductance, rate of leaf senescence, the relative water content, leaf area index, root system, and yield-related traits (Ravi et al., 2013; Megha et al., 2016). Leaf area index and root system play a particularly important role in times of stress. As mentioned above, genotypes with high root volume and low leaf area are more drought tolerant. GS could then predict the tolerance of cultivars to drought through the prediction of the potential of rooting, root development, and low leaf growth of the latter. However, the reduction of leaf area is not advantageous for yield because it impacts photosynthetic activity. Vanhove et al. (2012) therefore suggested the selection of vigorous plants that tend to reduce their growth only during mild drought stress. In addition, the research program on RTB at Consultative Group for International Agricultural Research (CGIAR) mentioned high-speed phenotyping tools such as the use of remote sensing, ground penetrating radar, near-infrared, electronic data collection, and image-based analysis that should be applied to obtain drought-related data efficient to GS (Friedmann et al., 2018). Similarly, Mastrangelo et al. (2012) reported several approaches for successful phenotyping of GS candidates. For complicated traits such as root systems and stomatal conductance, they can be independently assessed on the basis of their correlation with less difficult to assess traits.

Like drought, phenotyping BSD requires a good appreciation of the disease symptoms on the host plant and an effective descriptor. The area under the disease progression curve, the disease severity index, the rank of the youngest spotted leave, and the rank of the youngest attacked leaves are generally the appropriate notation to phenotype banana for BSD. The cultivars Bluggoe, Fougamou, Cachaco, and Lep Chang Kut are drought tolerant, whereas the cultivars IDN 077, Lagun Vunalir, Pisang Kelat and Pelipita are BSD resistant. The mechanisms of their resistance at the genomic level may involve the multivariate GS, especially by predicting the performance of the accessions in the field *via* indirect selection in the nursery. This indirect selection will then phenotype for traits that can be measured in the field such as physiological traits exhibited by the resistant cultivars. The majority of banana cultivars, particularly the plantain group, contain the genome B. From a genetic point of view, genome B brings rigidity to the fruit and resistance to cultivars against biotic and abiotic threats such as BSD and drought (Hamon and Nicolas, 1997). A phenotyping of cultivars with the B genome makes it possible to provide phenotypic data reflecting the global potential of GS on all the cultivated genotypes and to therefore conduct an inclusive study. As there is a challenge for obtaining a good training material in banana population, the collection of germplasm can be used (**Figure 6**). It will then be necessary initially to study the structure of this population using the SNPs obtained following treatment with tools specific to polyploids that have been presented in the previous sections. This study of the structure will make it possible to form a good TP and to develop an effective model that will be used for the selection of the breeding material.

## Conclusion

BSD and drought are the major threats to banana production, a polyploid crop that plays an important role in food security of thousands of consumers in several countries. We have shown the contribution of conventional breeding approaches in controlling these two threats in banana production. The few varieties obtained by varietal creation require a lot of time, and the costs of phenotyping over a long period are enormous and not accessible to all. GS has already shown its potential on several diploid crops, but its implementation in polyploid plants first experienced a lack of interest, undoubtedly because of the genomic complexity of polyploid crops. Indeed, the first genotyping platforms and the statistical methods used in GS were developed for diploid plants and therefore are ineffective for their polyploid counterparts. These tools generally do not take into account the complex genetic interactions encountered in polyploid crops. It should also be noted that many polyploid plants are clonally propagated and therefore are not conducive to obtaining a good TP.

The means used in artificial intelligence with ML and in bioinformatics with polyploid-specific programs are good prospects for implementing GS in polyploid plants. These new tools consider complex genetic interactions and increase the accuracy of selection. Investigations on these means would certainly make it possible to make GS effective on polyploid plants.

## Author contributions

LMN and EA-D designed, planned, and wrote the paper. DC and HN edited and wrote the paper. CA, NF, and JB checked and commented the final version. All authors listed approved the final version of the paper for submitting.

## Funding

The first author is a PhD candidate under the scholarship awarded by the Intra-Africa Academic Mobility Program (GENES) of the European Union at the University of Abomey-Calavi (Republic of Benin).

## Acknowledgments

We gratefully acknowledge the European Union and the African Union for supporting our study through the GENES Intra-Africa Academic Mobility project.

## Conflict of interest

The authors declare that the research was conducted in the absence of any commercial or financial relationships that could be construed as a potential conflict of interest.

## Publisher’s note

All claims expressed in this article are solely those of the authors and do not necessarily represent those of their affiliated organizations, or those of the publisher, the editors and the reviewers. Any product that may be evaluated in this article, or claim that may be made by its manufacturer, is not guaranteed or endorsed by the publisher.
